# Synthetic long non-coding RNAs [SINEUPs] rescue defective gene expression *in vivo*

**DOI:** 10.1038/srep27315

**Published:** 2016-06-06

**Authors:** Alessia Indrieri, Claudia Grimaldi, Silvia Zucchelli, Roberta Tammaro, Stefano Gustincich, Brunella Franco

**Affiliations:** 1 Telethon Institute of Genetics and Medicine (TIGEM), 80078, Via Campi Flegrei 34, Pozzuoli (NA), Italy; 2 Area of Neuroscience, SISSA, 34136, Trieste, Italy; 3 Dipartimento di Scienze della Salute, Universita’ del Piemonte Orientale, Novara, Italy; 4 Department of Neuroscience and Brain Technologies, Italian Institute of Technology, 16163, Genova, Italy; 5 TransSINE Technologies, 1-7-22 Suehiro-cho Tsurumi-ku, Yokohama, Kanagawa 230-0045 Japan; 6 Medical Genetics Services, Department of Translational Medicine, Federico II University, 80131, Naples, Italy

## Abstract

Non-coding RNAs provide additional regulatory layers to gene expression as well as the potential to being exploited as therapeutic tools. Non-coding RNA-based therapeutic approaches have been attempted in dominant diseases, however their use for treatment of genetic diseases caused by insufficient gene dosage is currently more challenging. SINEUPs are long antisense non-coding RNAs that up-regulate translation in mammalian cells in a gene-specific manner, although, so far evidence of SINEUP efficacy has only been demonstrated in *in vitro* systems. We now show that synthetic SINEUPs effectively and specifically increase protein levels of a gene of interest *in vivo*. We demonstrated that SINEUPs rescue haploinsufficient gene dosage in a medakafish model of a human disorder leading to amelioration of the disease phenotype. Our results demonstrate that SINEUPs act through mechanisms conserved among vertebrates and that SINEUP technology can be successfully applied *in vivo* as a new research and therapeutic tool for gene-specific up-regulation of endogenous functional proteins.

Both naturally occurring and artificial RNAs have the potential to be used as modulators of target genes. Regulation of gene expression through the activity of gene-specific artificial inhibitory nucleic acids such as siRNAs, RNAi and morpholinos (MOs) has become a common strategy to investigate gene function and they have also extended the druggable genome to potentially all protein coding genes. An equally important approach with potentially broad-range applications could be based on natural and artificial RNAs that can increase expression of target genes and few examples have been described. For instance, degradation or inhibition *in vivo* of natural antisense transcripts (NATs) by single-stranded oligonucleotides or siRNAs can transiently and reversibly modulate locus-specific gene expression^1^ and chemically modified mRNAs are capable to modulate gene expression in a mouse model of a lethal congenital lung disease^2^. Finally, a programmable transcription factor prototype has been shown to promote gene transcription in cell lines and primary cultures via an invariable transactivating domain coupled with a variable RNA domain that binds genes using sequence specificity^3^.

Large genomic efforts such as ENCODE^4^ and FANTOM^5^ have shown that the majority of the mammalian genome is transcribed. In addition to approximately 25000 protein-coding genes, there are at least an equal number of long non-coding RNA (lncRNA) genes that generate long transcripts (over 200 base pairs) that do not encode for proteins. About one third of annotated lncRNAs overlaps with protein-coding genes and many of these are transcribed from the opposite strand forming sense/antisense (S/AS) pairs^6^.

We have previously shown that a natural lncRNA antisense to the Ubiquitin carboxyl-terminal esterase L1 (AS *Uchl1*), a Parkinson’s disease-associated gene, is able to increase UchL1 protein synthesis at the post-transcriptional level^7^. We demonstrated that AS *Uchl1* activity depends on two distinct RNA elements, The Binding Domain (BD) at the 5′ end, is a sequence that overlaps, in antisense orientation, to the sense protein-coding mRNA and determines target selection and AS *Uchl1* specificity by RNA-RNA base pairing. The AS *Uchl1* BD is 72bp long, centered across the initiating ATG with a −40/+32 configuration, spans part of the *Uchl1* 5′UTR and a portion of its coding sequence (CDS)^7^. The other functional part of the AS *Uchl1* sequence is represented by the Effector Domain (ED), an inverted SINE (short interspersed nuclear elements) B2 sequence embedded in the non-overlapping part of the transcript that is essential for protein synthesis up-regulation^7^. Its modular architecture allows to redirect translation enhancement activity to any target mRNA by swapping its BD with the appropriate antisense sequence^8^. The AS *Uchl1* domain organization is conserved in other AS lncRNAs that overlap with protein coding genes and regulate their translation^7^,^9^. Therefore, AS *Uchl1* represents a new class of natural and synthetic antisense lncRNAs that can activate translation^7^,^10^. These RNAs were named SINEUPs because they require the inverted SINEB2 sequence to UP-regulate translation in a gene-specific manner^10^. Synthetic SINEUPs have been proven effective *in vitro* with a number of targets, including GFP^7^,^10^, FLAG-tagged proteins^10^ and secreted recombinant antibodies^9^, thus supporting the intrinsic scalability of SINEUP technology^10^.

However, to date SINEUP efficacy has only been limited to measuring target protein levels *in vitro* in mammalian cells, in experiments carried out in controlled, homogenous cell culture systems.

Here we show for the first time that SINEUPs can increase the synthesis of a functional endogenous protein *in vivo* and rescue haploinsufficient gene dosage in a medakafish model of a human disease.

Our study demonstrates that SINEUPs may be an effective tool for functional studies *in vivo* that demand increased levels of endogenous proteins. In addition, these results are a proof of principle that this technology may be applicable to design therapeutic approaches for genetic diseases in which selectively increasing the expression of the target gene may be curative.

## Results and Discussion

To assess the potential application of the SINEUP technology *in vivo,* we used the teleost medakafish (*Oryzias latipes*), which is particularly amenable for reverse-genetic analyses. In this model, the microinjection of early embryos with any kind of RNAs is technically easy and results in transient gene overexpression or inhibition^11^.

**Figure 1:**
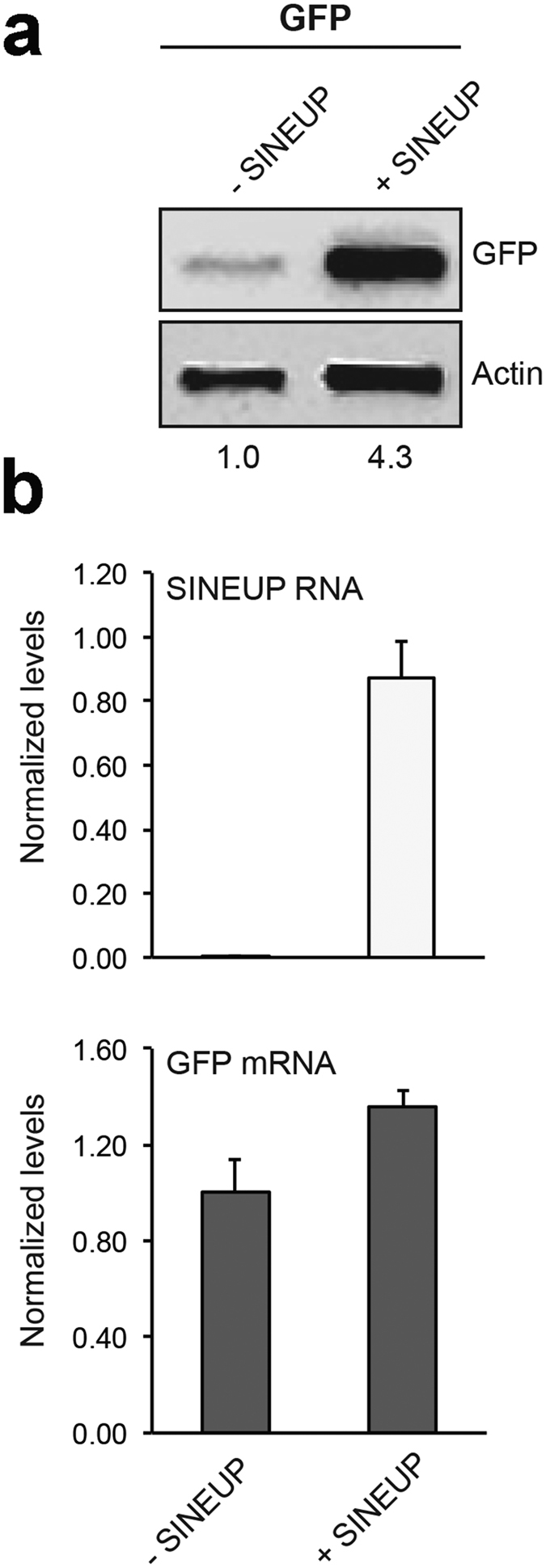
pCS2 + -SINEUP-GFP activity *in vitro*. HEK293T/17 were transfected with pEGFP-C2 and pCS2+/SINEUP-GFP constructs at 1:6 ratio (+SINEUP). Control cells were transfected with pEGFP-C2 and an empty control plasmid (-SINEUP). 48 hr after transfection, cells were lysed and processed for protein (**a**) and RNA (**b**) levels. Western blot was performed with anti-GFP antibody. β-actin was used as loading control. Fold-induction was calculated on Western blot images normalized to β-actin and relative to empty control samples. Expression of SINEUP-GFP (white bars) and quantity of GFP mRNA (grey bars) were monitored by Real Time PCR using specific primers. Data indicate mean ± SD. Data are representative of >3 independent replicas.

**Figure 2:**
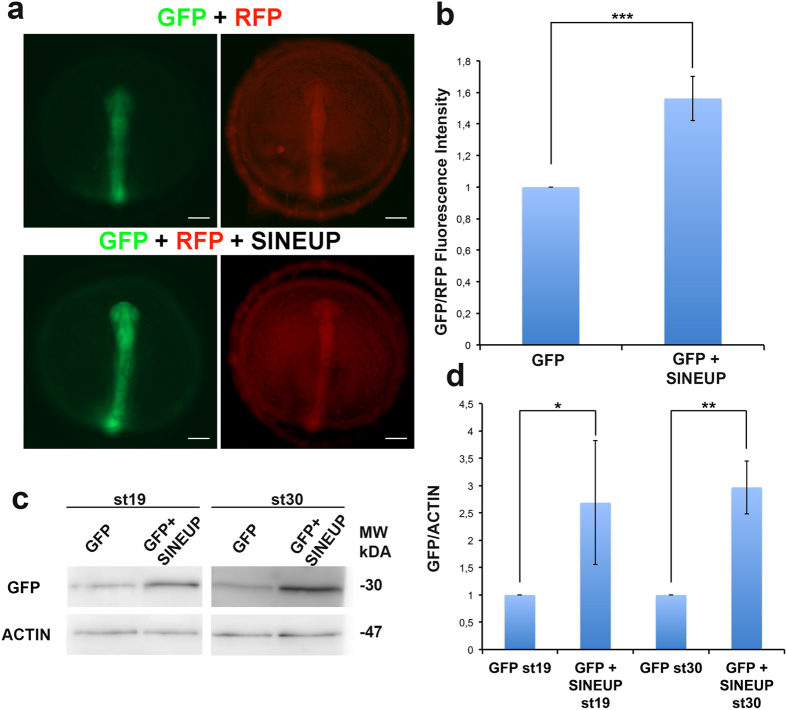
Synthetic SINEUP increases GFP protein levels *in vivo.* (**a**) Representative st19 embryos injected with RFP and GFP mRNA with or without SINEUP-GFP RNA. Scale bar 100 μm. (**b**) Quantification of GFP/RFP fluorescence intensity by ImageJ analysis software (n = 10, ***p < 0,001, one-tailed Student’s t-test; Error bars are SEM). (**c**) WB analysis of GFP in st19 and st30 embryos injected as in (**a**). (**d**) Quantification of GFP protein levels (n = 2, *p < 0,05, **p < 0,01, one-tailed Student’s t-test; Error bars are SEM).

For our initial studies we utilized the SINEUP-GFP^7^ that results in an increase GFP protein levels in transient overexpression experiments. SINEUP-GFP was cloned into the pCS2 plasmid that allows *in vitro* synthesis of RNA. As expected, we observed that in human embryonic kidney (HEK) 293T/17 cells, the SINEUP-GFP pCS2 construct increased GFP protein levels by acting post-transcriptionally (Fig. 1). To test their functionality *in vivo*, SINEUP-GFP RNA and GFP mRNA were transcribed in *vitro* and then co-injected into medaka embryos. Equal amounts of RFP transcribed mRNA were also co-injected as control for SINEUP specificity. SINEUP activity was estimated as fold change in GFP protein levels, normalized for RFP protein levels, in the presence or absence of the SINEUP. Embryos injected with the SINEUP-GFP displayed increased GFP levels and unchanged RFP levels (Fig. 2a,b). Furthermore, western blot (WB) analysis confirmed increased GFP levels at 19 and 30 stage (st) embryos (Fig. 2c,d). These results demonstrate the efficacy of SINEUPs and validate the post-transcriptional mechanisms of SINEUPs *in vivo.* SINEB2 sequences are mouse specific and are not present in fish, in which a superfamily of vertebrate SINEs (V-SINEs) has been described^12^. Our results indicate that SINEUPs act through mechanisms conserved among vertebrates and suggest that the secondary structure of SINEs is functionally conserved between mice and fish.

To address the potential use of this technology in increasing expression of an endogenous protein *in vivo* we tested SINEUPs in a medakafish model of microphthalmia with linear skin defects (MLS) syndrome, a X-linked dominant disorder characterized by microphthalmia, brain abnormalities and skin defects in heterozygous females, and *in utero* lethality in males^13^. MLS syndrome is caused by mutations in players of the mitochondrial respiratory chain (MRC) such as the holocytochrome c-type synthase (*HCCS*)^14^, and the subunit 7B of cytochrome c oxidase (COX), the MRC complex-IV^15^.

**Figure 3:**
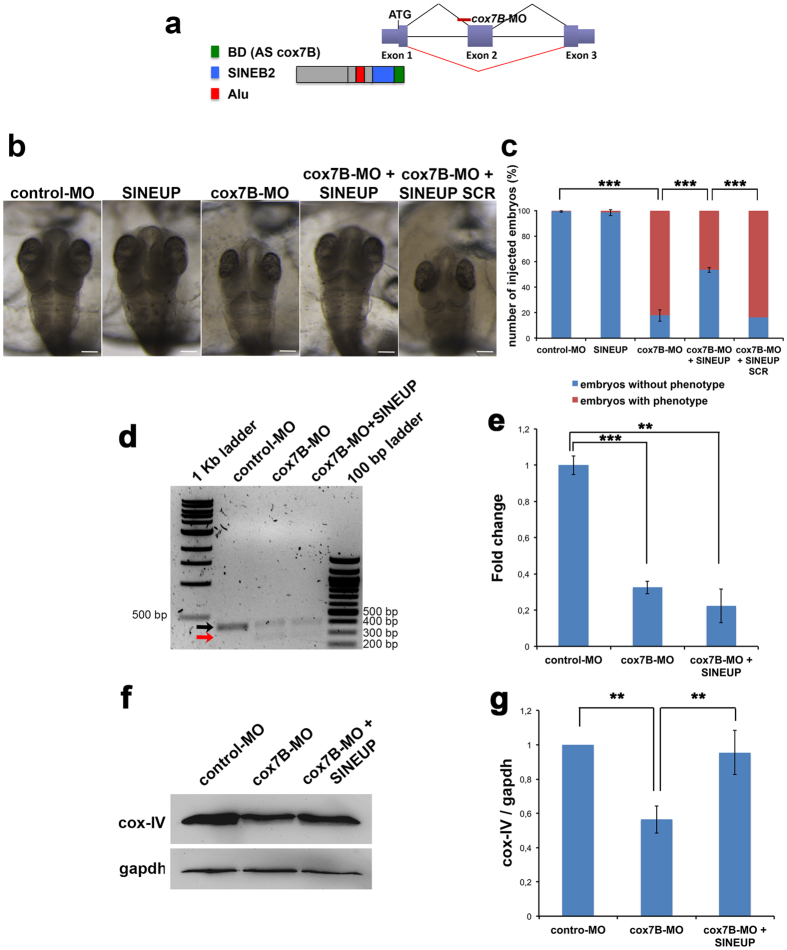
Synthetic SINEUP-cox7B rescues MLS phenotype in medakafish. (**a**) Structure of the medakafish *cox7B* transcript (exon/intron gene organization is displayed) and binding sites of cox7B-MO and SINEUP-cox7B. The cox7B-MO was designed against the acceptor splice site of exon 2 and its injection causes the skipping of exon 2. The BD of SINEUP-cox7B is designed against exon 1 in position −40 in the 5′-UTR (before ATG) to position +32 in the CDS. (**b**) Representative st30 embryos injected with control-MO, SINEUP-cox7B, cox7B-MO with or without SINEUP-cox7B or SINEUP-SCR. Scale bars 100 μm. SINEUPs rescue microphthalmia and microcephaly in 50% of cox7B-morphants (**c**). (n ≥ 300 embryos/conditions, ***p < 0,001, One-way ANOVA; Error bars are SEM). (**d**) RT-PCR of cox7B transcripts on total RNA extracted from embryos injected with control-MO, cox7B-MO and embryos co-injected with cox7B-MO and cox7B-SINEUP. Black arrow indicates WT mRNA whereas the red arrow indicates the mutated mRNA generated by cox7B-MO-driven aberrant splicing. (**e**) Real Time PCR on total RNA using specific primers to amplify WT *cox7B* mRNA. (n = 3 independent biological samples, ***p < 0,001, **p < 0,01, two-tailed Student’s t-test; Error bars are SEM). (**f**) WB analysis of cox-IV in st30 embryos injected with control-MO and cox7B-MO with or without SINEUP-cox7B. (**g**) Quantification of cox-IV protein levels (n = 3, **p < 0,01 one-tailed Student’s t-test; Error bars are SEM).

We downregulated *cox7B* expression using a MO-based approach. cox7B-MO was designed against the exon 2 acceptor splice-site and its injection causes exon 2 skipping resulting in frameshift^15^ (Fig. 3a). We designed a synthetic SINEUP against the endogenous *cox7B* mRNA carrying a 72bp BD starting from position −40 in the 5′UTR (before ATG) to position +32 in the CDS (Fig. 3a). We then cloned the SINEUP-cox7B sequence in the pCS2 plasmid and injected the *in vitro* synthesized RNA into embryos. As expected, injection of SINEUP-cox7B did not change *cox7B* mRNA levels (Supplementary Fig. 1a) and did not induce any aberrant phenotype in control embryos (Fig. 3b,c). cox7B-morphants showed a dose-dependent phenotype characterized by microcephaly and microphthalmia^15^ (Fig. 3b,c). Interestingly, injection of the SINEUP-cox7B in MLS morphants fully rescued microphthalmia and microcephaly in about 50% of embryos, whereas the injection of a control SINEUP carrying a scrambled sequence in the BD domain (SINEUP-SCR) did not result in amelioration of the phenotype (Fig. 3b,c).

To exclude that SINEUP could induce changes in *cox7B* transcription and/or interfere with MO functioning in cox7B morphants, we performed RT-PCR to amplify full-length WT and mutant *cox7B* mRNAs from cox7B-MO injected embryos and embryos co-injected with cox7B-MO and SINEUP-cox7B. Target sequences of cox7B-MO and SINEUP-cox7B are localized in different exons of *cox7B* and do not overlap (Fig. 3a). As expected, SINEUP-cox7B did not alter size or abundance of WT and mutated cox-7B mRNA (Fig. 3d,e) thus confirming that SINEUPs act through a post-transcriptional mechanism.

COX7B is necessary for complex-IV formation and its downregulation *in vitro* induces reduction of fully assembled complex-IV and of its subunits including COX-IV^15^ (Supplementary Fig. 2). As expected WB analysis showed reduction of cox-IV in cox7B-MO-injected fish (Fig. 3f,g). Interestingly, SINEUP-cox7B was capable to fully rescue cox-IV levels in cox7B-morphants whereas no effect was detected using the SINEUP-SCR in cox7B morphants (Fig. 3f,g and Supplementary Fig. 3) or the SINEUP-cox7B in wt embryos (Supplementary Fig. 1b,c).

**Figure 4:**
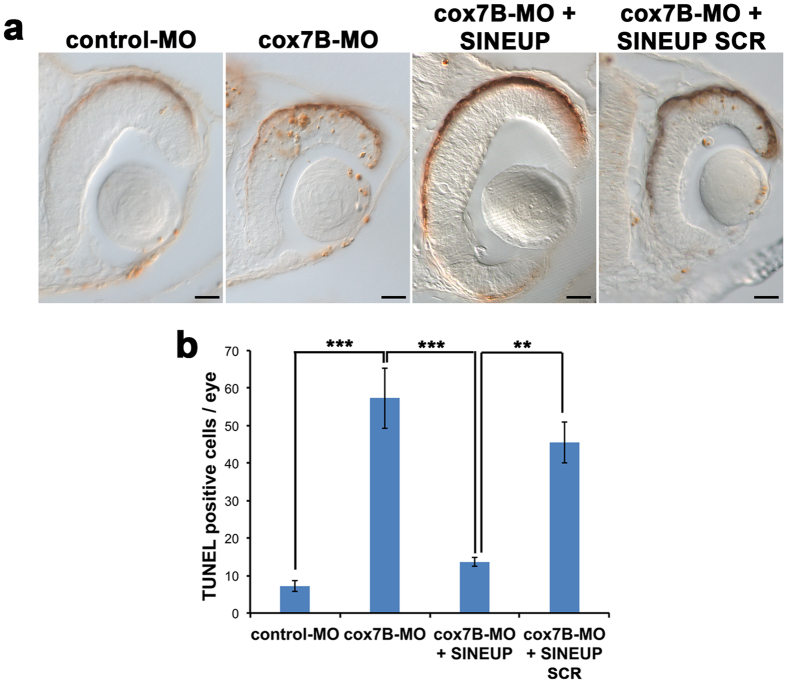
c*ox7B* downregulation induces increased cell death that is rescued by synthetic SINEUP-cox7B. (**a**) TUNEL assays on retinal sections of st30 embryos injected with control-MO, cox7B-MO with or without SINEUP-cox7B or SINEUP-SCR. Scale bars 20 μm. (**b**) Number of TUNEL positive cells/eye (n ≥ 5 retina/condition, **p < 0,01, ***p < 0,001, One-way ANOVA; Error bars are SEM).

We previously demonstrated that, in *hccs*-morphants, MRC impairment results in increased CNS programmed cell death (PCD) and that this event underlies the MLS phenotype^16^. Interestingly, TUNEL analysis revealed an increase of PCD in the eyes and brain of cox7B morphants, which was rescued by injection of SINEUP-cox7B (Fig. 4a,b).

Our data demonstrated that synthetic SINEUP-*cox7B* may restore MRC function thus consequently rescuing the MLS phenotype. Our results validate SINEUPs as a versatile tool for *in vivo* experimental biology and pave the way for its use in RNA therapeutics.

Artificial siRNAs, RNAi and MOs have become the tools of choice to inhibit gene expression and SINEUPs represent their molecular counterparts to increase protein synthesis *in vivo*. To our knowledge, SINEUPs represent the only available tool that uses AS lncRNAs to enhance production of endogenous proteins acting on their cellular mRNAs. In this context, SINEUP technology represents a powerful tool in molecular biology to increase protein levels of the specific gene of interest *in vivo*. Given their mode of action, SINEUPs would allow a spatio-temporal regulation of protein quantities in a more physiological context, avoiding artifacts due to ectopic overexpression and targeting only cells that actively express mRNA of interest, while being ineffective if mRNA is not present or present in a mutated form. On the basis of our results, SINEUPs should be considered a new approach to treat conditions such as haploinsufficiencies due to a reduction to 50% or less of gene function in which rescue of the phenotype requires recovery of the protein levels. A large number of genetic disorders are due to reduced levels of protein products. For these conditions, an efficient SINEUP activity specific for the gene of interest would, in principle, be therapeutic. This approach presents several advantages over competing technologies: a) SINEUPs can modulate translation of target mRNAs without introducing stable genomic changes; b) the induced up-regulation of specific proteins is a physiological range (~2-fold) compared to most conventional gene replacement strategies; c) SINEUPs exert their function on target mRNAs under their physiological regulation thus limiting adverse influences on cells and tissues that do not express the target transcript^8^,^17^.

In conclusion our data demonstrate that SINEUPs can selectively increase the expression of an endogenous functional protein *in vivo* and may be applied as a new tool in molecular biology and for nucleic acids-based therapeutics.

## Methods

### Plasmids

pEGFP-C2 (Clontech), SINEUP targeting EGFP (here named SINEUP-GFP), and SINEUP containing a scramble sequence in the region of overlap (Binding Domain, BD) (here named SINEUP-SCR) have been previously described^7^.

SINEUP targeting medakafish *cox7B* mRNA (Accession Number HE717026) was constructed using pcDNA3-Δ5-AS*Uchl1* as backbone^7^. SINEUP-backbone lacks the BD to *Uchl1* and retains AS Uchl1 effector domain (inverted SINEB2 element) embedded in AS Uchl1 non-overlapping sequence containing the partial Alu sequence and the 3′ tail. SINEUP *cox7B*-specific BD (72bp) was designed, in antisense orientation, around the ATG of the protein-coding sequence with a −40/+32 organization. For antisense *cox7B*, the method of ‘annealing and primer extension’ of two 3′-end overlapping oligonucleotides was used to generate the 72-bp antisense *cox7B* overlap region. Annealed fragment was obtained with the following oligonucleotides:

**AScox7BFW** 5′-ATATCTCGAGATGTTAGCTGCAGCCTTTGCAAACCGGTACATGTTGTCTGGCCTTGTG-3′

**AScox7BRv** 5′-GAGAGATATCCCGTTCTTCAAGGGTATCTGAAGACACAAGGCCAGACAACATGTACC-3′

The annealed fragment was digested with XhoI and EcoRV and ligated into antisense pcDNA3-Δ5-AS*Uchl1*.

Full-length GFP from pEGFP-C2 plasmid and full-length SINEP-GFP, SINEUP-SCR and SINEUP-cox7B from pcDNA3 plasmids were cloned into with pCS2+ vector.

pCS2+/RFP (Red Fluorescent Protein) was already described^16^.

### Cell lines and transfection

Human Embryonic Kidney (HEK) 293T/17 and HeLa cells were obtained from ATCC and maintained in culture with Dulbecco’s Modified Eagle Medium (GIBCO) supplemented with 10% FBS (SIGMA) and 1% antibiotics (penicillin/streptomycin), as suggested by the vendor.

HEK 293T/17 cells were transfected with Fugene HD (Roche), with a 1:6 ratio between pEGFP-C2 vector and pCS2+/SINEUP-GFP plasmids and following manufacture’s instruction. Cells were collected at 48 hours after transfection and split in two samples for RNA extraction and Western Blot analysis, as previously described^10^.

To silence *COX7B,* Hela cells were transfected using siRNAs ON-TARGET against human *COX7B* and ON-TARGET Non-Targeting (Darmachon) to a final concentration of 50 nM using Interferin reagent as previously described^15^. Cells were collected at 72 hr after transfection for protein extraction and Western Blot analysis.

### Medakafish (*Oryzias latipes)* Stocks

Wild type *Oryzias latipes* of the cab strain were maintained in an in-house facility (28 °C on a 14/10 hr light/dark cycle). Embryos were staged as described^18^.

All studies on fish were conducted in strict accordance with the institutional guidelines for animal research and approved by the Italian Ministry of Health; Department of Public Health, Animal Health, Nutrition and Food Safety in accordance to the law on animal experimentation (article 7; D.L. 116/92; protocol number: 00001/11/IGB; approval date June 6, 2011). Furthermore, all animal treatments were reviewed and approved in advance by the Ethics Committee of the Institute of Genetics and Biophysics, IGB Animal House, (Naples, Italy).

### mRNAs and MOs Injections

RNAs of SINEP-GFP, SINEUP-SCR, SINEUP-cox7B, GFP and RFP were transcribed *in vitro* from pCS2+ plasmids using the SP6 mMessage mMachine kit (Ambion) according to the manufacturer’s instructions. Control-MO (5′-CCTACCTACACCACAACAGAAATAA-3′) and cox7B-MO (5′-CCGACCTGCACGACAACACAAAGAA-3′) were purchased from Gene Tools, LLC. MOs working concentrations, injection conditions and their specificity and efficiency were previously described^15^. MOs and RNAs were injected into fertilized embryos at the one/two-cell stage according to the following conditions: GFP mRNA 50 ng, RFP mRNA 50 ng, SINEUP-GFP 300 ng, control-MO and cox7B-MO 100 μM, SINEUP-cox7B and SINEUP-SCR 300 ng. At least 3 independent experiments were performed for each condition.

### mRNA expression analysis

Total RNA extraction from cells and embryos was done using RNeasy Mini kit (Quiagen) according to the manufacturer’s instructions. During the extraction protocol RNAs were digested with DNaseI (Qiagen) to remove DNA contaminating, according to the manufacturer’s instructions. At least 50 embryos were pooled in each assay. Total RNA was then reverse-transcribed into cDNA by QuantiTect Reverse Transcription Kit (Quiagen) with random hexamers oligo.

For RT-PCR experiment, full length cox7B transcripts were amplified from cDNAs of control-MO, cox7B-MO and cox7B-MO + SINEUP-cox7B using the following primers designed against the exon 1 and exon 3 respectively:

**cox7BEx1Fw** 5′-CGCAACCATTTCCGGTATCTG-3′

**cox7BEx3Rw** 5′-GGTATTGGTTTGTCATCTGTAC-3′

PCR fragments were loaded on 2% agarose gel and subjected to electrophoresis.

For quantitative real time PCR analysis (qRT-PCR), cDNAs was diluited 1:2 and analyzed using Roche Light Cycler 480 system with LigthCycler 480 SYBR Green I Master (Roche). Oligonucleotide sequences of *GFP* and human *GAPDH* primers were previously described^7^. To specifically amplify WT medakafish *cox7B* mRNA, we used the following primers designed against the exon 3 and exon 2 that is skipped after the MO-driven aberrant splicing^15^:

**cox7BEx2Fw** 5′-CAGTGGAACCGTCTTCTGC-3′

**cox7BEx3Rw** 5′-GGTATTGGTTTGTCATCTGTAC-3′

Medakafish *hprt* and *gapdh* primers were previously described^19^,^20^ and used as endogenous controls. mRNA analysis showed comparable results with both normalization approaches. Relative expression was calculated with the ΔΔCt method^21^ and variation was reported as fold change (2^−ΔΔCt^). The results are shown as means ± SEM of three independent biological assays.

### Protein extracts and Western blot analysis

Cells or embryos (at least 100 for conditions) were lysed in RIPA buffer containing 1X protease inhibitor cocktail (SIGMA). Cleared proteins extracts were quantified by using the Bradford method (Bio-Rad). For WB, protein samples were separated on 15% SDS-PAGE and transferred to PVDF membrane (Millipore). Membranes were blocked in TBS containing 5% Non Fat Dry Milk (Cell Signaling) and incubated with the follows primary antibodies: anti-GFP 1:500 (Abcam, ab13970), anti GFP rabbit polyclonal antibody (Life technologies, a6445), used 1:1000 (for HEK 293T/17 cells experiments), anti-β-Actin 1:500 (Sigma), anti-GAPDH 1:1000 (Santa Cruz Biotechnology), anti-COX-IV 1:500 (Cell Signaling), anti-COX7B 1:500 (Abcam, ab137094).

Proteins of interest were detected with horseradish peroxidase-conjugated goat anti-mouse or anti-rabbit IgG antibody (1:3000, GE Healthcare) and rabbit anti-chicken IgY antibody (Millipore) visualized with the Luminata Crescendo substrate (Millipore) or the Super Signal West Femto substrate (Thermo Scientific), according to the manufacturer’s protocol. Western blotting images were acquired using the Chemidoc-lt imaging system (UVP) and band intensity was calculated using ImageJ software.

### TUNEL assay

TUNEL assays were performed on st30 embryos using *In Situ* Cell Death Detection Kit (Roche, Mannheim, Germany) as previously reported^16^.

### Statistical Analysis

In all experiments the significance of differences between groups was evaluated by ANOVA and Student’s t-test, p < 0.05 was considered significant. Quantitative data are presented as the mean ± SD (Standard Deviation) or ± SEM (Standard Error of the Mean) of at least three independent experiments.

## Additional Information

**How to cite this article**: Indrieri, A. *et al*. Synthetic long non-coding RNAs [SINEUPs] rescue defective gene expression *in vivo. Sci. Rep.* **6**, 27315; doi: 10.1038/srep27315 (2016).

## Supplementary Material

Supplementary Information
