# Supplementary material for: Synthetic long non-coding RNAs [SINEUPs] rescue defective gene expression in vivo
Source: Sci Rep. 2016 Jun 6;6:27315. doi: 10.1038/srep27315 (PMC4893607; doi:10.1038/srep27315)
Supplement: Supplementary Information [file srep27315-s1.pdf]

SUPPLEMENTARY INFORMATION

Synthetic long non-coding RNAs [SINEUPs] rescue defective gene expression *in vivo*

Alessia Indrieri, Claudia Grimaldi, Silvia Zucchelli, Roberta Tammaro, Stefano Gustincich, Brunella Franco.

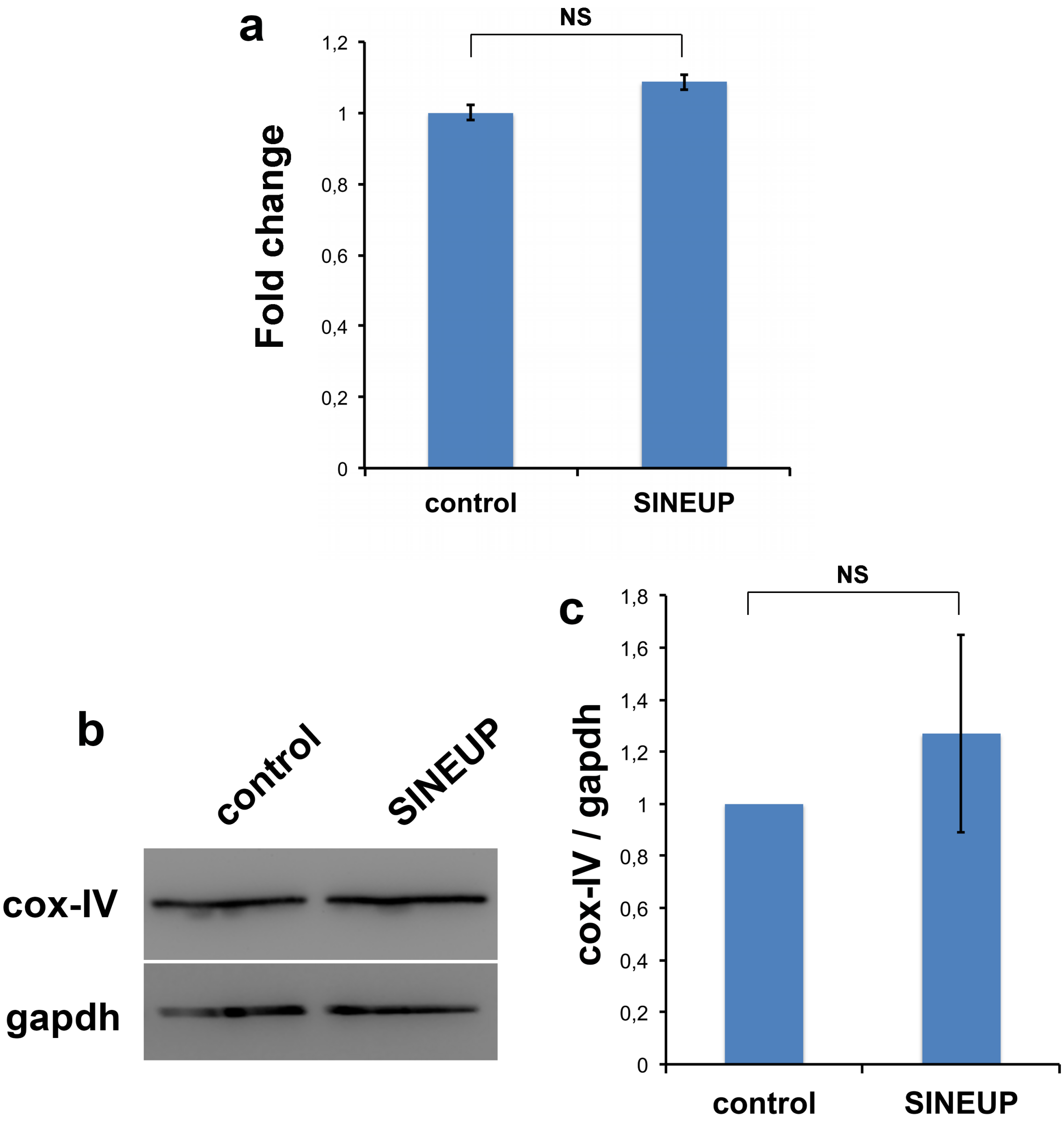

Supplementary Figure S1. SINEUP-cox7B does not change *cox7B* mRNA and COX-IV protein levels in wt (control) embryos. (a) Real Time PCR on total RNA from st30 wt embryos using primers that amplify *cox7B* mRNA. (n=3 independent biological samples, Error bars are SEM). (b) WB analysis of cox-IV in st30 embryos injected with SINEUP-cox7B. gapdh was used as loading control. (c) Quantification of cox-IV protein levels (n=3 independent biological samples; Error bars are SEM). NS = not significant

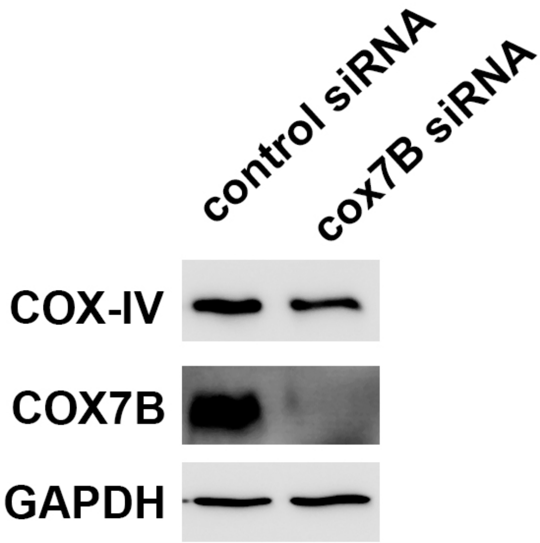

Supplementary Figure S2. COX7B downregulation reduces COX-IV levels in HeLA cells. WB analysis of COX-IV in HeLa cells transfected with control siRNA and COX7B siRNA.

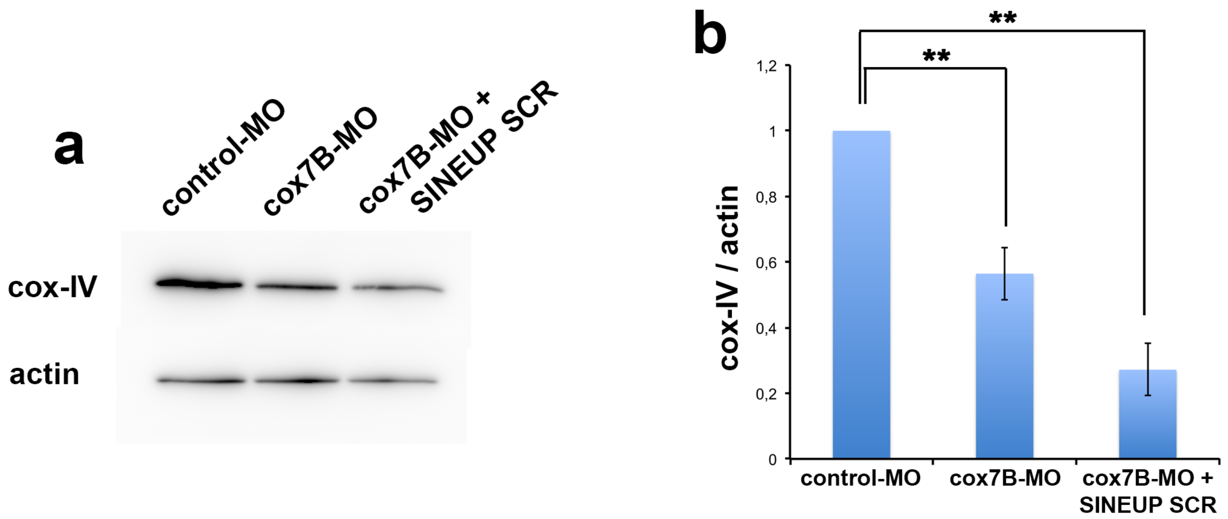

Supplementary Figure S3. SINEUP-SCR does not influence cox-IV levels. (a) WB analysis of cox-IV in st30 embryos injected with control-MO, cox7B-MO with or without SINEUP-SCR. (b) Quantification of cox-IV protein level (n=3, ** $p<0,01$, one-tailed Student's t-test; Error bars are SEM).
